# Predictive value of the pendulum test for assessing knee extensor spasticity

**DOI:** 10.1186/s12984-018-0411-x

**Published:** 2018-07-18

**Authors:** Alyssa Whelan, Andrew Sexton, Melony Jones, Colleen O’Connell, Chris A. McGibbon

**Affiliations:** 1 Institute of Biomedical Engineering, University of New Brunswick, Fredericton, NB E3B 5A3 Canada; 2 Faculty of Kinesiology, University of New Brunswick, Fredericton, NB Canada; 3 Stan Cassidy Centre for Rehabilitation, Fredericton, NB Canada

**Keywords:** Spasticity, Knee extensor, Knee flexor, Pendulum test, Relaxation index, Modified Ashworth scale, Classification, Logistic regression, Receiver operator characteristic

## Abstract

**Background:**

The pendulum test is commonly used to quantify knee extensor spasticity, but it is currently unknown to what extent common pendulum test metrics can detect spasticity in patients with neurological injury or disease, and if the presence of flexor spasticity influences the test outcomes.

**Methods:**

A retrospective analysis was conducted on 131 knees, from 93 patients, across four different patient cohorts. Clinical data included Modified Ashworth Scale (MAS) scores for knee extensors and flexors, and years since diagnosis. BioTone™ measures included extensor strength, passive and active range of motion, and pendulum tests of most affected or both knees. Pendulum test metrics included the relaxation index (RI), 1st flexion amplitude (F1amp) and plateau angle (Plat), where RI=F1amp/Plat. Two-way ANOVA tests were used to determine if pendulum test metrics were influenced by the degree of knee flexor spasticity graded by the MAS, and ANCOVA was used to test for confounding effects of age, years since injury, strength and range of motion (ROM). In order to identify the best pendulum test metrics, Receiver Operator Characteristic analysis and logistic regression (LR) analysis were used to classify knees by spasticity status (none or any) and severity (low/moderate or high/severe).

**Results:**

Pendulum test metrics for knee extensors were not influenced by degree of flexor spasticity, age, years since injury, strength or ROM of the limb. RI, F1amp and Plat were > 70% accurate in classifying knees by presence of clinical spasticity (from the MAS), but were less accurate (< 70%) for grading spasticity level. The best classification accuracy was obtained using F1amp and Plat independently in the model rather than using RI alone.

**Conclusions:**

We conclude that the pendulum test has good predictive value for detecting the presence of extensor spasticity, independent of the existence of flexor spasticity. However, the ability to grade spasticity level as measured by MAS using the RI and/or F1amp may be limited. Further study is warranted to explore if the pendulum test is suitable for quantifying more severe spasticity.

## Background

Muscle spasticity can be a painful and debilitating complication that negatively impacts function and quality of life in people with upper motor neuron injury from neurological disease or trauma [1], such as acquired brain injury (trauma, stroke), cerebral palsy, multiple sclerosis and spinal cord injury. Management of spasticity typically involves pharmacologic intervention and/or ongoing physical therapy [2, 3], but a significant barrier to effective treatment prescription is the inability to quantify spasticity in the clinic [4].

Spasticity is typically assessed by inducing a rapid stretch of the muscle, or administering a “stretch-reflex” test. Clinical tests such as the Modified Ashworth Scale [5] and Tardieu Scale [6] apply this method to quantify spasticity subjectively, but their inter-rater reliability [7–10] and validity [11] have been questioned. Several studies have examined objective approaches to quantifying spasticity in the clinic using wearable sensor technologies during passive muscle stretches [9, 12–15], but there is not yet a clear consensus on testing protocol and how to best translate the resulting electrophysiological and biomechanical signals into clinically relevant measures of spasticity. As such, the Modified Ashworth Scale remains a commonly used method of quantifying spasticity in clinical settings.

For the knee joint, the Wartenberg pendulum test [16] offers a potential solution for translation to clinical assessment. The pendulum test offers a simple approach whereby gravity induces the stretch-reflex of knee muscles by dropping the lower-leg from a resting horizontal position, and observing its oscillatory behavior throughout the passive movement [17, 18]. The test’s easy implementation and execution with commonly available sensors (e.g. electromyography with video [19], goniometry [20, 21], and other accessible devices such as the Wii remote [22]) has contributed to it emerging as an objective and reliable way to quantify spasticity in the knee extensors [17, 23–25]. Nevertheless, there is still a lack of consensus on what pendulum test metrics are most relevant to clinical spasticity assessment, and importantly, whether the pendulum test is sensitive to knee flexor spasticity. Thus the purpose of this study was two-fold:

1. To determine if the measurement of extensor spasticity is influenced by flexor spasticity during the pendulum test in patients with neurological injury or disease; and
2. To determine which pendulum test metrics are the closest indicators of clinical muscle spasticity, as represented by the Modified Ashworth Scale (MAS).

## Methods

This is a retrospective analysis of knee spasticity measurement data from a multi-site study to evaluate wearable sensor-based systems for acquiring objective measures of muscle tone in the clinic.

### Participants

#### Ethics, consent and screening

The study was approved by the University Research Ethics Board (REB) and by the REB or Institutional Review Board (IRB) for each of three participating clinical sites. The three clinical sites were medium to large rehabilitation hospitals in eastern Canada (Stan Cassidy Centre for Rehabilitation, Fredericton, New Brunswick, and Nova Scotia Rehabilitation Hospital, Halifax, Nova Scotia) and U.S. (Spaulding Rehabilitation Hospital, Boston, Massachusetts). Prior to data collection, all participants in the study provided informed, signed consent. Data were collected at the three sites between September 2011 and May 2014.

The site coordinator (non-therapist) approached patients meeting the inclusion criteria and asked if they were willing to participate in the study during a future regularly scheduled visit. Those who were willing were informed of the study purpose, risks, discomforts, potential benefits, and their rights to privacy and the use of data. Once a participant agreed to the outlined procedures and written consent was obtained, they were enrolled in study. Additional details of the larger study can be found elsewhere [13].

*Inclusion criteria were*: Male or female active inpatient or outpatient, sixteen years of age or older, and currently receiving services at study site for one or more of the following diagnoses: acquired brain injury (ABI: strokes, trauma, etc.), spinal cord injury (SCI: incomplete any level or complete C7 and below), multiple sclerosis (MS: meeting 2010 MacDonald criteria [26]), and cerebral palsy (CP: hemiplegic or diplegic); medically stable; and exhibits some degree of abnormal tone in either upper or lower limbs, specifically at the elbow and/or knee joint(s). *Exclusion criteria were*: Joint conditions such as osteoarthritis, rheumatoid arthritis, etc. that would confound measurement of spasticity; bariatric or with little measureable surface EMG signal; viral or bacterial infection; open skin lesions, and; not capable of autonomous consent.

### Measurements

Clinical assessment was performed on the patient as regularly scheduled. This included manual assessment of knee extensors and flexors, where the therapist used the Modified Ashworth Scale (MAS). The MAS is a ubiquitous instrument for clinical spasticity assessment, in which the therapist performs a manual stretch-reflex and rates the patient’s spasticity on a 6-point scale with categories 0, 1, 1+, 2, 3 and 4, as defined elsewhere [5]. There were no participants with MAS = 4, which by definition is indicative of full rigidity [5] and cannot be assessed with a pendulum test.

The participant’s age, height, weight, leg length (for the pendulum test), diagnosis (ABI/CVA, MS, CP, or SCI), affected extremity (right, left or both), and the month and year of onset or injury, were also recorded. The participant was then assessed using the BioTone™ system, as follows.

#### Active and passive knee extension

**Fig. 1:**
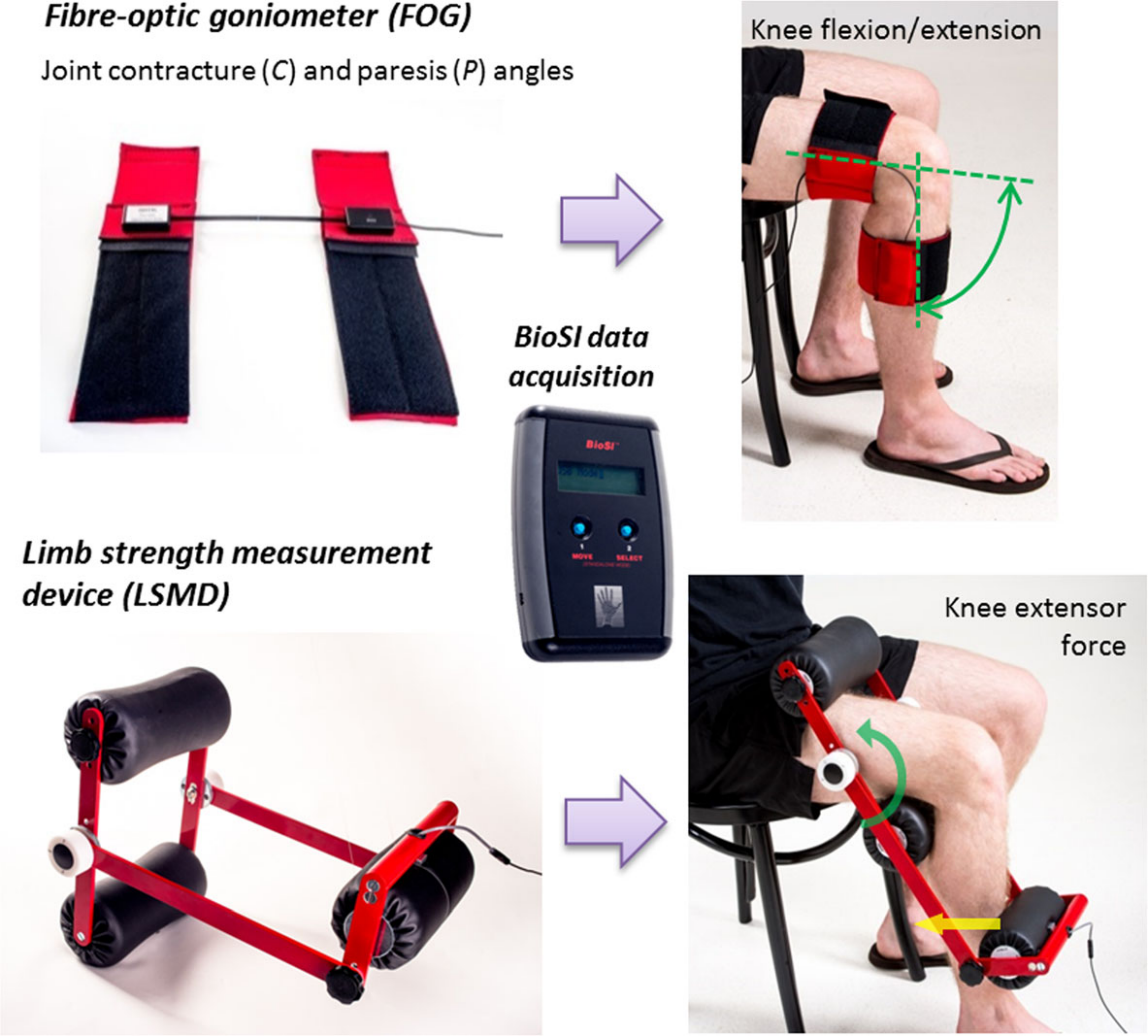
BioTone™ toolkit for lower-extremity assessment of range of motion and strength. Top: ShapeSensor™ (Measureand Inc.) fibre-optic goniometer (FOG) is inserted into Neoprene and Velcro mounting cuffs, and attached to the leg to measure knee flexion/extension angle. Bottom: Limb strength measurement device (LSMD) is shown for the knee in extensor configuration. Centre: BioSI (University of New Brunswick, Canada) data acquisition unit used to acquire sensor data and save it to the laptop computer

While seated, a fibre-optic goniometer (FOG, ShapeSensor™, Measurand, Fredericton NB) was positioned on the limb using the Neoprene/Velcro cuffs and secured in place as seen in Fig. 1 (top). For passive knee extension, the therapist moved the participant’s limb slowly to the full extension. For active knee extension, the subject was instructed to move their lower leg against gravity from a flexed to fully extended position. For both active and passive tests, the minimum angle achieved (peak extension angle) was recorded and used to estimate the degree of contracture and paresis.

#### Isometric muscle strength

Knee extensor strength was measured with the limb strength measurement device (LSMD) shown in Fig. 1 (bottom). The LSMD was designed to enable autonomous muscle force measurement, thus eliminating the variability inherent with hand-held dynamometry protocols or subjectivity of the manual muscle test. The LSMD was adjusted to “extensor” orientation and positioned on the leg such that the subject’s knee joint formed an angle of approximately 90 deg. The subject then sat with leg supported (by therapist) and attempting to extend the knee with maximal effort. The load cell in the LSMD recorded the patient’s maximal force generation. This was repeated three times with 15 s between trials. Because the “lever arm” of the LSMD (distance from joint centre to distal pad which contains the force transducer) was fixed, forces measured by the device are a proportional measure of muscle torque across participants, and were normalized by dividing by body weight.

#### Pendulum test

For the instrumented pendulum test, the FOG was positioned as described above. To avoid interference with the thigh cuffs, EMG electrodes were placed on the lateral vastii and lateral hamstring. The reference electrode was placed on the hand. The participant was positioned in a reclining wheelchair with legs hanging freely over the edge of the seat and torso inclined to approximately 30 degrees to avoid stretching the biceps femoris. The therapist then slowly raised the lower leg to full extension (or passive extension limit) and held the leg horizontal until the participant was completely relaxed, as indicated by real-time EMG display. The participant’s lower-leg was then released and allowed to oscillate until coming to rest (oscillation amplitude less than 3 deg). Pendulum tests were repeated at least three times for the right and/or left legs. In the present study, EMG was only used to confirm if a spastic contraction occurred during the test.

### Data analysis

**Fig. 2:**
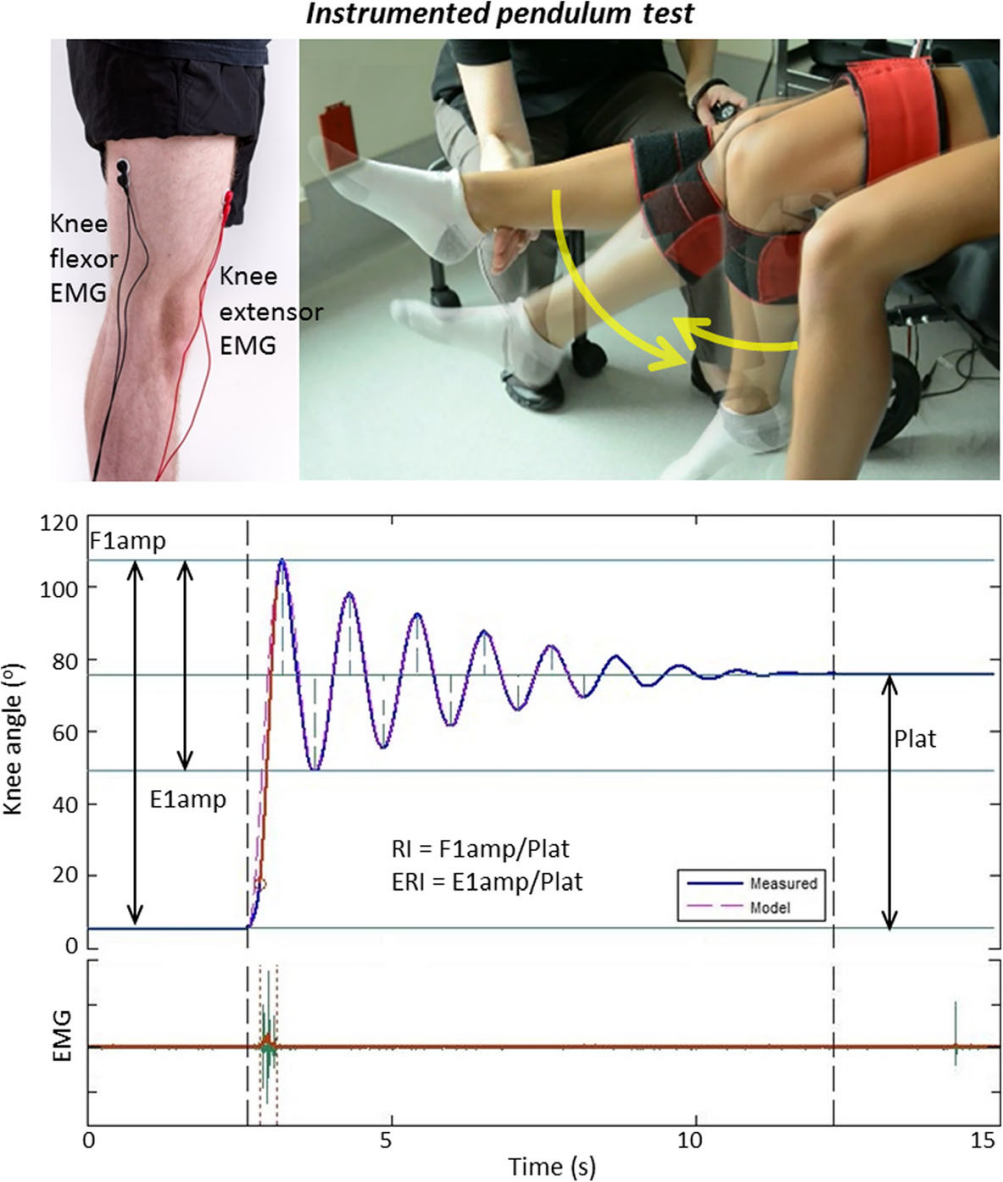
BioTone™ toolkit for lower-extremity assessment of spasticity using the pendulum test. Top: Electromyographic (EMG) electrodes are placed on extensor and flexor muscles and used to ensure muscle relation prior to the start of testing and to monitor involuntary contractions during the test, and fibre-optic goniometer (FOG) system used to measure knee angle during the pendulum test. Bottom: Pendulum test metrics include the plateau angle (Plat), first flexion amplitude (F1amp), and first extension amplitude (E1amp) which are used to compute the relaxation index (RI) and the extension relaxation index (ERI)

Pendulum test metrics (RI, F1amp, Plateau angle, etc.) extracted for analysis have been described in detail elsewhere [19] and are shown in Fig. 2. Number of cycles (full oscillations) was counted between start of motion and until the oscillation amplitudes is less than 3 degrees [27]. Data from knees of participants with bilateral involvement were treated as separate data points. All statistical analyses were completed using IBM Statistical Package for Social Science (IBM SPSS, Version 23). All statistical analyses were conducted with an alpha level of .05 for statistical significance.

First we used a 2-way ANOVA to compare pendulum test metrics (RI, F1amp, E1amp, ERI, Plateau angle and Number of cycles as described in Fig. 2) across levels of extensor and flexor spasticity, coded as binary independent variables from the clinical MAS scores. Binary variables were required for testing classification models as part of the second objective.

We created two separate models in order to determine if the above metrics can classify patients by no or any spasticity, and whether we can classify present spasticity as low or high. In the first model (Model 1), we divided knees into no spasticity (MAS = 0) and any spasticity (MAS > =1), based on their clinical assessment of knee extensor and flexor spasticity prior to the pendulum test. In the second model (Model 2), we excluded the MAS = 0 knees and re-grouped knees by low/moderate spasticity (MAS = [1,1+]) and high/severe spasticity (MAS= [2, 3]), for extensors and flexors. Although other groups could be used, the selected groupings had the best distribution and were considered the most clinically relevant.

Therefore, Model 1 had factors: *Ext1* (0 = No extensor spasticity, 1 = Any extensor spasticity) by *Flx1* (0 = No flexor spasticity, 1 = Any flexor spasticity), and Model 2 had factors: *Ext2* (0 = Low/moderate extensor spasticity, 1 = High/severe extensor spasticity) by *Flx2* (0 = Low/moderate flexor spasticity, 1 = High/severe flexor spasticity). Models were tested for main effects of extensor spasticity and flexor spasticity, and interaction effects between extensor and flexor spasticity. Interaction effects were used to answer the first research question, and the main effects analysis was used to answer the second research question, as detailed below:

#### Flexor spasticity influence on measurement of extensor spasticity

To determine if the measurement of extensor spasticity is influenced by flexor spasticity during the pendulum test we need to analyze the interaction effects of the ANOVA tests for Model 1 and Model 2. A significant interaction effect for Model 1 would suggest that *any* flexor spasticity might influence the outcome of the extensor spasticity assessed using the pendulum test. A significant interaction effect for Model 2 would suggest that the *amount* of flexor spasticity may be an important consideration for trusting the measurement of extensor spasticity using the pendulum test. These analyses were then repeated with a variety of covariates (age, time since diagnosis/injury, passive and active extension range and isometric extensor strength) to further explore sources of variability in the pendulum test.

#### Assessment of pendulum test metrics for quantifying spasticity

To determine which pendulum test metrics are the best indicators of clinical muscle spasticity as represented by the Modified Ashworth Scale (MAS), we first need to analyze the main effects of the ANOVA tests for Model 1 and Model 2. A significant main effect for extensor (or flexor) spasticity will indicate if the pendulum test metric is sensitive to spasticity state of the knee. For pendulum test metrics with significant main effects, we then determined which metric offered the best predictive value of clinical spasticity. This was accomplished using Receiver Operator Characteristic (ROC) analysis to determine which metrics have the best predictive potential (area under curve, AUC > .7), followed by classification analysis using logistic regressions on the binary spasticity groupings (Model 1 and Model 2) for each of the pendulum test metrics. Tests for Model 1 will determine which metric is best at discriminating any spasticity from no spasticity, and the analysis of Model 2 will determine which metric is best at grading spasticity into low/moderate spasticity versus high/severe spasticity.

## Results

Ninety-three patients (65 male and 28 female) composed of four patient cohorts (45 with ABI, 14 with MS, 12 with CP, and 22 with SCI) completed the pendulum test assessments and had MAS scores and other measures available. A total of 53 patients were measured unilaterally and 39 bilaterally, resulting in a total of 131 knees tested (56 ABI, 23 MS, 18 CP and 34 SCI). Participant demographic data (age and years since diagnosis) for the four cohorts are shown in Table 1. Also included are BioTone™ measures of contracture (passive extension range), paresis (passive – active extension range) and strength (peak isometric extensor force) for each cohort group.

**Table 1:** Participant demographic data and BioTone™ assessment of contracture, paresis and strength

	Gender		Age, years		Years Since Dx		Contracture (Passive ROM Min, deg)		Paresis (Passive ROM min- Active ROM min, deg)		Flexor Strength (N)		Knees
	M	F	Mean	(SD)	Mean	(SD)	Mean	(SD)	Mean	(SD)	Mean	(SD)	N
	n	n											
ABI (*n* = 45)	35	10	52	(16)	4.8	(4.8)	−6.96	(5.25)	7.46	(12.9)	258.1	(135.7)	56
MS (*n* = 14)	6	8	54	(13)	17.2	(8.7)	−5.26	(4.81)	11.3	(19.2)	215.7	(134.2)	23
CP (*n* = 12)	5	7	34	(12)	34.3	(11.9)	−4.28	(7.12)	6.50	(5.93)	160.8	(45.9)	18
SCI (*n* = 22)	19	3	45	(14)	6.4	(10.5)	−6.11	(5.17)	13.3	(20.2)	153.1	(99.4)	34
Total (*n* = 93)	65	28	49	(16)	11.0	(12.9)	−6.09	(5.46)	9.52	(15.7)	215.7	(126.4)	131

*AB* acquired brain injury including stroke; *MS* multiple sclerosis; *CP* cerebral palsy; *SCI* spinal cord injury

MAS score for knee flexors and extensors for patient cohorts are shown in Table 2, and are grouped according to most affected and least affected side, with the total number of knees shown in the last row. The majority of flexor knees were assigned a score of 1 (*n* = 31) or 1+ (*n* = 24). Likewise, extensor knees followed a similar pattern with 33 knees being assigned a grade of 1, and 14 being assigned a grade of 1+, but with 16 being assigned a grade of 2. A total of 46 and 53 knee flexors and extensors, respectively, had a score of 0.

**Table 2:** Modified Ashworth Scale scores for knee flexors and extensors from clinical examination

	MAS score – Knee flexors					MAS score – Knee extensors				
	0	1	1+	2	3	0	1	1+	2	3
	n	n	n	n	n	n	n	n	n	n
*Most affected side*										
ABI (*n* = 34)	20	8	2	2	2	14	11	4	4	1
MS (*n* = 17)	6	2	4	5	0	6	1	3	4	3
CP (*n* = 11)	3	3	1	1	3	0	4	1	3	3
SCI (*n* = 20)	6	5	3	2	4	7	8	3	2	0
Total (*n* = 82)	35	18	10	10	9	27	24	11	13	7
*Least affected side*										
ABI (n = 22)	6	10	4	1	1	8	8	3	1	2
MS (*n* = 6)	2	1	2	0	1	2	0	2	1	1
CP (*n* = 7)	2	2	3	0	0	3	0	3	1	0
SCI (n = 14)	3	0	5	5	1	5	4	2	1	2
Total (*n* = 49)	13	13	14	6	3	26	9	3	3	4
Total (*n* = 131)	46	31	24	16	12	53	33	14	16	11

*ABI* acquired brain injury including stroke; *MS* multiple sclerosis; *CP* cerebral palsy, *SCI* spinal cord injury

**Fig. 3:**
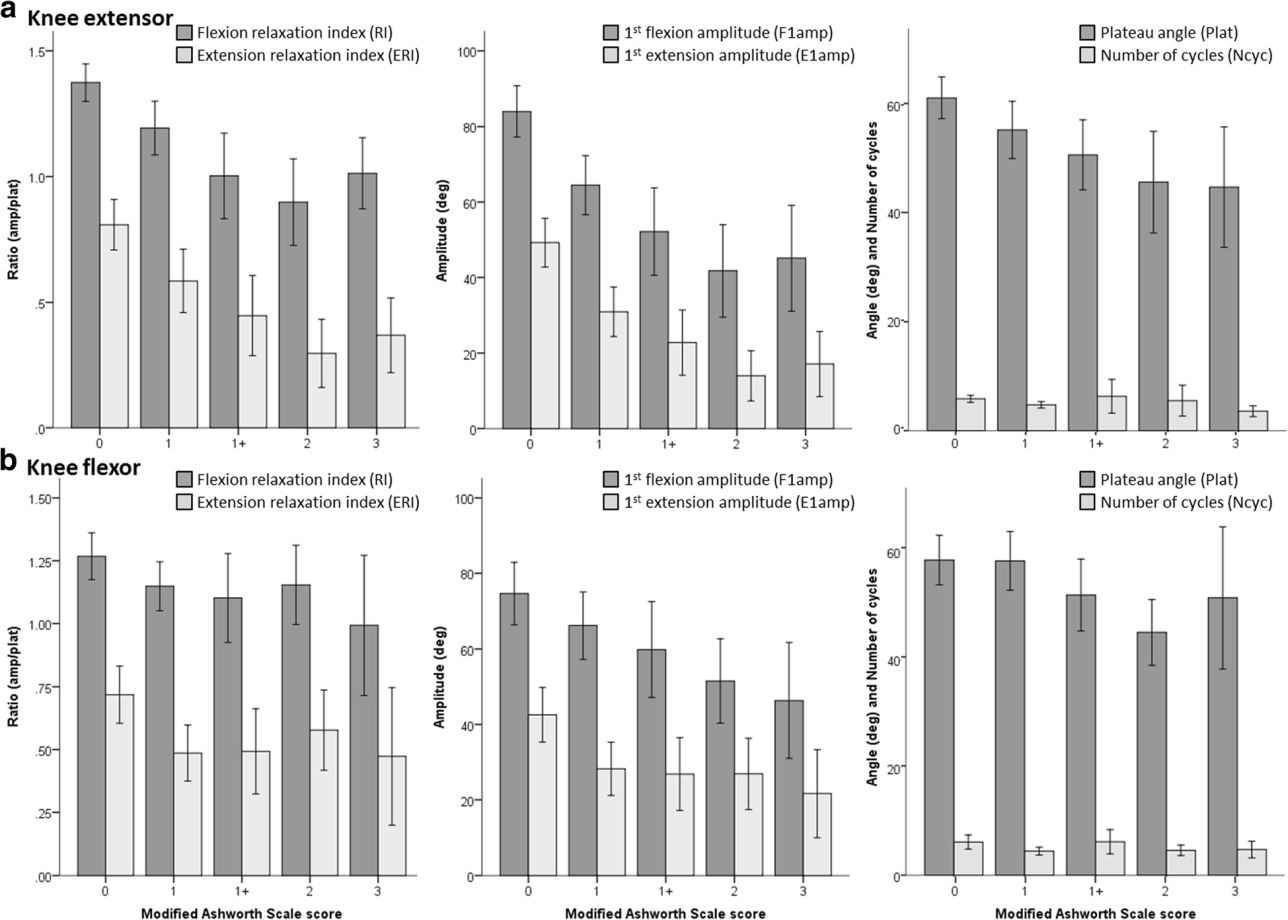
Spasticity metrics derived from the instrumented pendulum test for with standard deviation error bars for **a**. knee extensors and **b**. knee flexors. Left side panels show mean scores by MAS category flexion relaxation index (RI) and extension relaxation index (ERI) phases. Middle panels show mean scores by MAS category for 1st flexion amplitude (F1amp) and 1st extension amplitude (E1amp) phases. Right side panels show mean scores by MAS category for plateau (final resting) angle (Plat) and number of oscillation cycles (Ncyc)

Pendulum test outcomes are summarized in Fig. 3, and Tables 3 and 4. Figure 3 shows pendulum test metrics against therapist rated MAS scores for knee extensors (top) and flexors (bottom). Data in Tables 3 and 4 show the marginal means of the pendulum test scores according to the factorial levels of each model. Table 3 shows results for Model 1 with factors *Ext1* and *Flx1*, and Table 4 shows results for Model 2 with factors *Ext2* and *Flx2*. Table 5 shows the resulting *p*-values generated for main effects and interaction effects from testing Model 1 (top) and Model 2 (bottom).

**Table 3:** Model 1 marginal means for factors *Ext1* and *Flx1* on pendulum test metrics (RI = relaxation index, ERI = extension relaxation index, F1amp = 1st flexion amplitude, E1amp = 1st extension amplitude, Plat = plateau angle, and Ncyc = number of oscillation cycles)

MODEL 1		*Flx1*: Flexor spasticity					
		No spasticity (MAS = 0)			Any spasticity (MAS > 0)		
*Ext1*: Extensor spasticity							
		Mean	(SD)	n	Mean	(SD)	n
RI	No spasticity	1.4	(.2)	26	1.3	(.3)	19
	Any spasticity	1.1	(.3)	22	1.1	(.3)	64
ERI	No spasticity	.9	(.3)	26	.7	(.3)	19
	Any spasticity	.5	(.4)	22	.4	(.3)	64
F1amp	No spasticity	88.2	(22.6)	26	78.2	(21.5)	19
	Any spasticity	58.6	(26.7)	22	52.8	(24.6)	64
E1amp	No spasticity	54.6	(21.6)	26	41.9	(19.6)	19
	Any spasticity	28.3	(21.0)	22	22.1	(17.5)	64
Plat	No spasticity	62.4	(13.3)	26	59.1	(11.8)	19
	Any spasticity	52.2	(16.7)	22	49.4	(16.0)	64
Ncyc	No spasticity	6.3	(2.1)	26	5.2	(2.2)	19
	Any spasticity	5.8	(6.3)	22	4.9	(3.5)	64

**Table 4:** Model 2 marginal means for factors *Ext2* and *Flx2* on pendulum test metrics (RI = relaxation index, ERI = extension relaxation index, F1amp = 1st flexion amplitude, E1amp = 1st extension amplitude, Plat = plateau angle, and Ncyc = number of oscillation cycles)

MODEL 2		*Flx2*: Flexor spasticity					
		Low spasticity (MAS = [1,1+])			High spasticity (MAS= [2, 3])		
*Ext2*: Extensor spasticity							
		Mean	(SD)	n	Mean	(SD)	n
RI	Low spasticity	1.1	(.3)	30	1.1	(.5)	9
	High spasticity	.8	(.4)	8	1.0	(.3)	17
ERI	Low spasticity	.5	(.3)	30	.6	(.5)	9
	High spasticity	.2	(.1)	8	.4	(.3)	17
F1amp	Low spasticity	60.8	(24.5)	30	48.1	(22.2)	9
	High spasticity	42.9	(31.9)	8	45.9	(19.3)	17
E1amp	Low spasticity	24.4	(19.2)	30	28.0	(20.7)	9
	High spasticity	12.1	(13.1)	8	19.5	(12.8)	17
Plat	Low spasticity	53.6	(14.4)	30	47.8	(15.1)	9
	High spasticity	47.1	(21.0)	8	43.8	(16.0)	17
Ncyc	Low spasticity	4.7	(2.7)	30	5.2	(1.8)	9
	High spasticity	6.9	(7.7)	8	4.1	(1.8)	17

**Table 5:** Significant levels for 2-way ANOVA tests for pendulum test metrics

*p*-values	RI	ERI	F1amp	E1amp	Plat	Ncyc
Model 1						
*Ext1*	< 0.001*	< 0.001*	< 0.001*	< 0.001*	0.001*	0.594
*Flx1*	0.264	0.057	0.098	0.013*	0.304	0.181
*Ext1* × *Flx1*	0.679	0.520	0.654	0.395	0.948	0.956
Model 2						
*Ext2*	0.103	0.021*	0.149	0.040*	0.251	0.598
*Flx2*	0.319	0.031*	0.477	0.269	0.319	0.262
*Ext2* × *Flx2*	0.238	0.827	0.259	0.704	0.776	0.093

*effect is significant at *p* < .05

Model 1: Ext1 and Flx1 are coded 0 = No spasticity vs 1 = Any spasticity

Model 2: Ext2 and Flx2 are coded 0 = Low/ moderate spasticity vs 1 = High/ severe spasticity

### Does flexor spasticity influence the pendulum test?

Statistical results in Table 5 for analysis of Model 1 revealed a significant effect of *Ext1* for all metrics (*p* < 0.001), except for Number of cycles (*p* = 0.594). No significance effect of *Flx1* was present for any metrics except for E1amp (*p* = 0.013). Additionally, there were no significant *Ext1* × *Flx1* interactions (*p* > 0.05). For Model 2, the only pendulum test metrics to show any statistically significant differences were ERI, which had a significant *Ext2* effect (*p* = .012) and *Flx2* effect (*p* = .031), and E1amp which had a significant *Ext2* effect. Similar to Model 1, there were no significant (*p* > .05) interactions observed for any of the pendulum test metrics.

Lastly, ANCOVA were conducted using both models to identify any confounding effects of age, time since injury/diagnosis, passive ROM, active-passive ROM, and extensor strength. We found that both models maintained the same significance patterns when covariates were entered into the model. Therefore, covariates did not confound any effects on pendulum test metrics.

These results show that the pendulum test metrics studied were not significantly influenced by level of spasticity in the antagonistic flexor muscles, and were unaffected by variability in the covariates listed above. In addition, results showed that the pendulum test metrics studied had a good ability to discriminate between no spasticity and any spasticity of knee extensors, as grouped by the assigned MAS. However, they were not able to discriminate very well between low/moderate and high/severe spasticity.

### Which pendulum test metric(s) best predicts clinical spasticity?

**Fig. 4:**
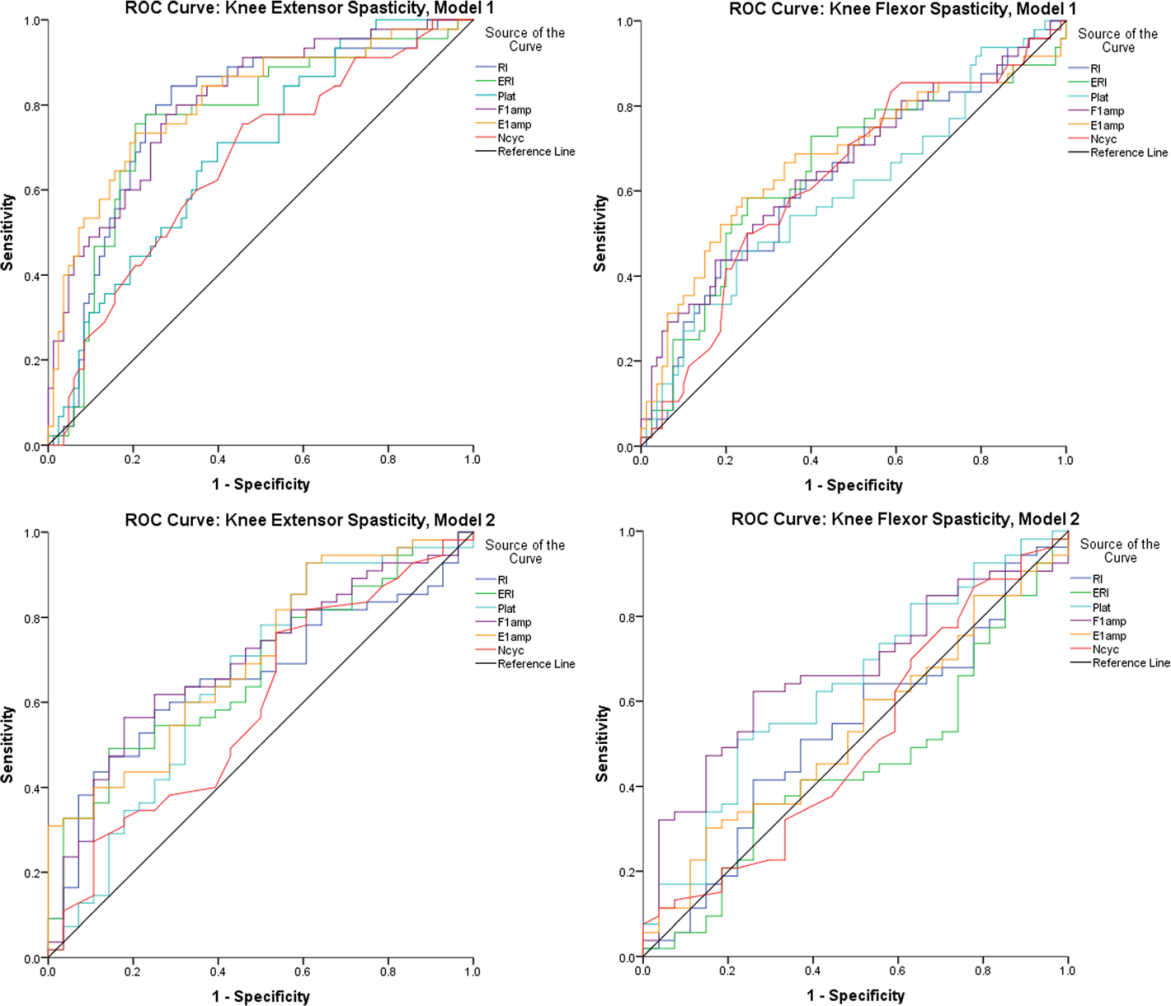
Receiver Operator Characteristic (ROC) curves for Model 1 (top panels) and Model 2 (bottom panels) for extensors (left panels) and flexors (right panels), for each of the pendulum test metrics studied

We first conducted ROC analysis on each of the metrics to evaluate their ability to detect the presence of spasticity (Model 1) and level of spasticity (Model 2). AUC values from operator curves (in Fig. 4.) are presented in Table 6. AUC values >.7 indicate the metric is potentially a good predictor of the dichotomous dependent variable, and AUC of ≤.5 indicates no predictive potential (diagonal line in ROC graphs in Fig. 4).

**Table 6:** ROC analysis area under curve (AUC) values for each pendulum test metric and for Model 1 and Model 2 prediction of spasticity

AUC (95% CI)	RI	ERI	F1amp	E1amp	Plat	Ncyc
Model 1						
*Ext1*	0.784 (0.698,0.869)	0.762 (0.672,0.852)	0.807 (0.729,0.885)	0.808 (0.726,0.890)	0.691 (0.599,0.783)	0.665 (0.567,0.762)
*Flx1*	0.635 (0.534,0.736)	0.652 (0.549,0.754)	0.655 (0.554,0.756)	0.675 (0.573,0.778)	0.600 (0.496,0.70)	0.631 (0.530,0.731)
Model 2						
*Ext2*	0.659 (0.541,0.777)	0.675 (0.557,0.792)	0.695 (0.578,0.812)	0.705 (0.589,0.820)	0.655 (0.523,0.786)	0.590 (0.459,0.721)
*Flx2*	0.526 (0.392,0.661)	0.446 (0.314,0.580)	0.665 (0.544,0.785)	0.531 (0.400,0.662)	0.636 (.508,0.764)	0.506 (0.368,0.644)

For Model 1, most metrics were acceptable predictors of *Ext1*, having AUC > .7, with the exception of Number of cycles and Plateau angle which had AUC’s of 0.66 and 0.69, respectively. Not surprisingly, Model 1 yielded poor results for predicting *Flx1* (Factors MAS = 0, and MAS = 1,1+,2,3) with AUC values < 0.7 for all pendulum test metrics. Model 2 yielded AUC values between 0.59 and 0.71 for predicting *Ext2* (Factors MAS = 1,1+ and MAS = 2,3) and all were < .7 for predicting *Flx2* (Factors; MAS = 1,1+, and MAS = 2,3). This analysis suggests that RI, ERI, F1amp, E1amp and (borderline) Plateau angle are individually acceptable predictors of extensor spasticity as graded by the MAS, but with no clear winner.

Given that E1amp and ERI are somewhat redundant in their nature of depicting contraction of the flexor muscles, and the above results suggest they have limited predictive value, both were dropped from the remaining analysis. The metrics RI and F1amp appear to be the superior metrics for predicting spasticity presence, per any MAS greater than 0. Clearly RI is a function of F1amp, and Plateau angle, as shown in Fig. 2. Therefore, we next conducted logistic regression analysis to classify extensor muscle spasticity according to Model 1 (*Ext1*) and Model 2 (*Ext2*) using RI alone, versus using F1amp and Plateau angle in the logistic regression.

These results are shown in Tables 7 and 8, respectively. For each metric the classification table is shown with resulting sensitivities and specificities as well as positive and negative predictive values (*PPV* and *NPV*) and overall classification accuracy. The logistic regression model for each metric is footnoted in the tables.

**Table 7:** Classification results using logistic regression for Model 1 prediction of extensor spasticity from pendulum test metrics: RI, F1amp, and Plat

LR terms for Model 1 (*Ext1*)	Observed *Ext1*	Predicted *Ext1*		Predictive value
		No spasticity	Any spasticity	
^a^RI	No spasticity	23	22	*NPV* = .51
	Any spasticity	12	74	*PPV* = .86
	Overall	*Spec* = .66	*Sens* = .77	*CA* = 74.0%
^b^F1amp	No spasticity	24	21	*NPV* = .53
	Any spasticity	14	72	*PPV* = .84
	Overall	*Spec* = .63	*Sens* = .77	*CA* = 73.3%
^c^Plat	No spasticity	15	30	*NPV* = .33
	Any spasticity	11	75	*PPV* = .87
	Overall	*Spec* = .58	*Sens* = .71	*CA* = 68.7%
^d^F1amp, Plat	No spasticity	28	17	*NPV* = .62
	Any spasticity	12	74	*PPV* = .86
	Overall	*Spec* = .70	*Sens* = .81	*CA* = 77.9%

^a^RI: Constant = 5.153; Beta_(RI)_ = −3.646, *p* < .001

^b^F1amp: Constant = 4.236; Beta_(F1amp)_ = −.051, *p* < .001

^c^Plat: Constant = 3.383; Beta_(Plat)_ = −.049, *p* < .001

^d^F1amp, Plat: Constant = 3.258; Beta_(F1amp)_ = −.073, *p* < .001; Beta_(Plat)_ = .045, *p* = .082

**Table 8:** Logistic regression results for Model 2 prediction of extensor spasticity from pendulum test metrics: RI, F1amp, and Plat

LR terms for Model 2 (*Ext2*)	Observed *Ext2*	Predicted *Ext2*		Predictive value
		Low spasticity	High spasticity	
^a^RI	Low spasticity	52	5	*NPV* = .91
	High spasticity	26	3	*PPV* = .10
	Overall	*Spec* = .66	*Sens* = .38	*CA* = 64.0%
^b^F1amp	Low spasticity	49	8	*NPV* = .86
	High spasticity	20	9	*PPV* = .31
	Overall	*Spec* = .71	*Sens* = .53	*CA* = 67.4%
^c^Plat	Low spasticity	54	3	*NPV* = .95
	High spasticity	22	7	*PPV* = .24
	Overall	*Spec* = .71	*Sens* = .70	*CA* = 70.9%
^d^F1amp, Plat	Low spasticity	50	7	*NPV* = .88
	High spasticity	20	9	*PPV* = .31
	Overall	*Spec* = .71	*Sens* = .56	*CA* = 68.6%

^a^RI: Constant = 1.006; Beta_(RI)_ = − 1.623, *p* = .026

^b^F1amp: Constant = .834; Beta_(F1amp)_ = −.029, *p* = .005

^c^Plat: Constant = 1.044; Beta_(Plat)_ = −.035, *p* = .022

^d^F1amp, Plat: Constant = .897; Beta_(F1amp)_ = −.028, *p* = .072; Beta_(Plat)_ = −.003, *p* = .907

For Model 1 (Table 7), the best classification accuracy value of 77.9% was for the model using F1amp and Plateau angle simultaneously, versus using any one of them individually or using the RI term alone. This model had the highest sensitivity (.81), specificity (.7), PPV (.86) and NPV (.62). For Model 2 (Table 8.) the highest classification accuracy value was 70.9% for the model using Plateau angle alone, followed by 68.6% for the model using F1amp and Plateau angle in combination. However, it is clear for Model 2 that predictively is heavily biased toward the negative condition; that is, the classifier was better at identifying negative results (low/moderate spasticity: MAS = [1,1+]) than positive results (high/severe spasticity: MAS= [2, 3]).

These results show that F1amp and Plateau angle explained more variance in the logistic regression analysis than RI alone, for detecting the presence of spasticity per a MAS or 1 or greater (*Ext1*). However, no pendulum test metrics were suitable for discriminating between knees with high/severe spasticity and those with low/moderate spasticity (*Ext2*) as measured by MAS.

## Discussion

The Wartenberg pendulum test [16] has been around since the 1950’s, but gained interest in the late 80’s/early 90’s as test for assessing muscle spasticity [17, 18, 23, 24]. Several studies have shown feasibility of using sensor technologies with an instrumented pendulum test [19, 21, 28] and repeatability and validity for assessing spasticity in patients has generally been reported as positive [25, 29, 30]. Although the test has some drawbacks [31], such as being sensitive to posture during the test [32, 33], clinical evidence is increasing that the pendulum test has value for objectively quantifying spasticity of knee extensors [34–39], as well as flexors and extensor of the elbow [40, 41].

Due to the velocity-dependent nature of spasticity, the initial and second swing of the pendulum test are considered key for the detection of spasticity [17]. The maximum angular velocity of the knee occurs during the initial flexion oscillation [19, 27]. This first excursion (F1amp) results in the largest velocity of musculotendinous stretch, and has been found to be sensitive to differences in spasticity in the quadriceps [23]. Bajd and Vodovnik [17] further refined the approach by dividing F1amp by the plateau angle to quantify the relaxation index, RI.^1^

Therefore, the RI is simply F1amp adjusted for the resting position Plateau angle (hence excursion angle), thus accounting for changes in muscle length and structure that can result from ongoing spasticity, or activity induced changes in resting tonic reflex. Stillman and McMeeken [19] introduced the extension relaxation index, or ERI, along with its characteristic amplitude E1amp, describing the excursion of the first (return) extension oscillation. In theory, this metric should be sensitive to flexor spasticity, although there is very little evidence of the use of ERI/E1amp in clinical studies.

Several studies have identified F1amp and/or RI [17, 23, 27, 34, 35] as acceptable metrics for identifying spasticity of the quadriceps, although its reliability has been debated in other works [27]. While these metrics have been identified as acceptable, many patient groups with knee extensor spasticity also experience knee flexor spasticity; in our study, MAS scores for extensors and flexors of patient’s knees were significantly correlated (*r* = .525, *p* < .001). To our knowledge, no other studies have examined the influence of flexor spasticity on the commonly reported pendulum test output metrics mentioned above. Our study provides convincing evidence that the degree of flexor spasticity does not have a significant impact upon commonly used pendulum test metrics F1amp and RI.

In addition, we quantified the predictive value of these metrics for detecting the presence of muscle spasticity, and for discriminating the level of spasticity as measured by MAS. The results demonstrate that the pendulum test is a valid tool to distinguish knee extensors with spasticity (MAS > 0), from those without spasticity (MAS = 0), but that none of the metrics we analyzed were able to discriminate between knees with low/moderate (MAS = [1,1+]) and high/severe (MAS= [2, 3]) spasticity. The data in Fig. 3 provide a potential explanation for this observation: note that RI and F1amp decreased in magnitude until extensor MAS = 2 then increase for MAS = 3 to levels similar as MAS = 1+. This U-shape tendency was similarly reported between RI and reflex torque from a model-based analysis [20], but was not observed in other studies [27].

Figure 3 also shows that Plateau angle had a more linear relationship with extensor MAS score. This may also explain, when classifying by Model 1 (no spasticity vs any spasticity), why the combination of F1amp and Plateau angle in the logistic regression model had better classification accuracy than did the models with RI, F1amp or Plateau angle individually. This supports the notion proposed by others [41] that F1amp corresponds to stretch-reflex hyper-excitability and Plateau angle corresponds to passive resistance to stretch of the muscle, both of which comprise (and confound [4]) the clinical presentation of spasticity when using the MAS. This finding is consistent with the study by Fowler et al. [41], who reported that variability in resting angle (our plateau angle) contributed to their finding of low reliability of the RI for children with CP; which adds further support to using F1amp and Plateau angle as separate independent variables for quantifying spasticity.

Although none of the metrics we studied passed muster for Model 2 classification (low spasticity vs high spasticity), the metric with the best overall accuracy, sensitivity and specificity (>.7) was Plateau angle, as shown in Table 8. However, the PPV was too low (.24) to be of value for reliably discriminating spasticity in the clinic, and it failed to reach AUC criteria (>.7) using the ROC analysis. It is important to note, however, that we only examined a few of the available metrics from the pendulum test. Other variables or approaches may be more sensitive to discriminating level of spasticity, such as time to first peak, first excursion velocity, and area under the pendulum curve [25, 42], stiffness and damping properties [20, 36, 43], or deriving metrics from combining kinematic information with electromyographic (EMG) information [44].

Other studies have reported that number of cycles is an indicator of spasticity [17, 41]. In our study, however, number of cycles had the poorest classification performance of all metrics for both Model 1 and 2 analyses. Although our averages for number of cycle agreed with data reported by others [41], Fig. 3 shows that the number of cycles did not correspond to MAS score.

### Limitations

This study had several limitations. Firstly, the gold standard used for comparison was the Modified Ashworth Scale (MAS), which despite its ubiquity has been questioned as a meaningful measure of spasticity due to the intermingling effects of hyper-excitability of the stretch reflex and passive resistance to stretch [4, 9]. This means that the inability of the pendulum test metrics to discriminate between low/moderate and high/severe spasticity in our study may have been due to subjectivity and lack of sensitivity of the MAS scale, rather than the ability of the pendulum test to quantify the effect. Nevertheless, data from others [41] shows that the pendulum test was less reliable when muscle becomes exceedingly resistant to passive stretch, which stands to reason given the limited excursion of the limb after release in patients with severe spasticity (c.f. [20, 25]).

Another limitation is that the study was carried out at three different hospitals across four different cohorts of patients. Regarding the different sites, the BioTone™ software fully controlled the testing protocol (from order of testing to acquisition of all measures) and all three sites used the same version of the system and sensors, and all site personnel (physical therapists) were trained by the research team (M.J. and A.S.) when the system was deployed. Of greatest concern, would be the variability across therapists in their MAS assessments, however, this was not analyzed in the current paper. Regarding patient groups, although we did not analyze effects by patient cohort (due to the lower numbers of MS and CP) the measurement approach (clinical MAS and BioTone) was independent of neurological etiology, and it is expected that the relationships between pendulum test metrics and clinical assessment would not be influenced by this fact.

## Conclusions

The pendulum test metrics studied for quantifying knee extensor spasticity were not sensitive to level of flexor spasticity (as measured by MAS), age, years since diagnosis, or physical parameters of the limb. The pendulum test metrics RI and F1amp were good predictors of the presence of clinical spasticity in knee extensors, however, none of the metrics studied were acceptable predictors of the level of spasticity, as measured by MAS. The best logistic regression model for detecting presence of spasticity used F1amp and Plateau angle as separate input terms rather than using RI or F1amp alone. More research is needed to determine if pendulum test metrics are sensitive outcomes measures for managing problematic spasticity.

## Footnotes

1 note that R2 was originally used by [17] but most authors now use RI as introduced by [19].
